# University teachers’ profiles based on digital innovativeness and instructional adaptation to COVID-19: Association with learning patterns and teacher demographics

**DOI:** 10.1007/s10639-023-11748-y

**Published:** 2023-04-11

**Authors:** Tahani Z. Aldahdouh, Mari Murtonen, Jere Riekkinen, Henna Vilppu, Trang Nguyen, Petri Nokelainen

**Affiliations:** 1 Faculty of Education and Culture, University of Tampere, Tampere, Finland; 2 Faculty of Education, University of Turku, Turku, Finland

**Keywords:** Digital innovativeness; Learning patterns, Pedagogical training, COVID-19 pandemic, University teachers; Instructional change

## Abstract

The purpose of this study was to understand the factors behind university teachers’ ability to implement instructional changes during the COVID-19 pandemic. An online questionnaire comprised of open-ended and Likert-scale questions was administered to teachers at a Finnish university in April 2020. The sample consisted of 378 university teachers who were categorised into four groups based on their digital innovativeness and the extent to which they implemented changes to adapt their teaching practices to COVID-19 restrictions: Avoider Survival Adapters, Avoider Ambitious Adapters, Embracer Survival Adapters, and Embracer Ambitious Adapters. We examined the association between the teacher groups and their learning patterns and background characteristics. The findings showed that Embracer Ambitious Adapters have significantly more meaning-oriented and application-oriented learning patterns than Embracer Survival Adapters, though Avoider Survival Adapters have more problematic learning patterns. Furthermore, the results indicated that pedagogical training and having more teaching experience helped innovative teachers embrace more changes in their teaching practices during the COVID-19 pandemic. In terms of discipline, the results showed that teachers working in hard disciplines (e.g., physics) were more likely to belong to the Embracer Survival Adapters group, while teachers working in soft disciplines (e.g., history) were more likely to belong to the Embracer Ambitious Adapters group. Possible interpretations of these findings and perspectives for further research are discussed.

## Introduction

Over two years have passed since the spread of the COVID-19 pandemic and the announcement of the immediate shift to online teaching at higher education institutions (HEIs) across the globe. Teachers, without prior notice, found themselves obliged to abandon their regular teaching practices, undergo key changes, adapt to the new normal and re-think their lesson planning, teaching and assessment methods. University teachers are continuously confronted with successive changes, such as those related to curriculum reform and digital transformation. Coping with the abrupt disruption brought by COVID-19 nevertheless increased the number of changes.

Recent studies have examined how university teachers experienced and responded to the sudden transition to online teaching during the pandemic. For instance, research has addressed teacher’s readiness (Scherer et al., 2021), perceptions (Almahasees et al., 2021), emotions (Meishar & Ariella, 2021), expectations, experiences and challenges (Marek et al., 2021; Mensa & Grow, 2020; Riekkinen et al., 2022; Spoel et al., 2020). Other studies have identified factors that contributed to teachers’ coping ability, including personal attributes, such as previous experience in online teaching (Meishar & Ariella, 2021; Scherer et al., 2021), positive attitudes towards technology (Spoel et al., 2020), feeling responsible for students’ learning (Marek et al., 2021) and professional identity (Bruggeman et al., 2021), as well as institutional attributes, such as technological support (Scherer et al., 2021), infrastructure (Mittal et al., 2021) and training (Oliveira et al., 2021). Although these studies advanced knowledge, much uncertainty still exists regarding factors particularly associated with teachers’ ability to implement instructional changes during the pandemic. One of the handful of studies done thus far is that of Lee & Jung (2021), which showed that university teachers’ technology acceptance and innovativeness are among the factors that most highly correlated with instructional change.

In this study, we assumed that innovativeness is a fundamental characteristic in studying teachers’ ability to cope with COVID-19 restrictions. Innovativeness refers to the degree to which a teacher is receptive to new ideas and makes innovation decisions independently of others (Midgley & Dowling, 1978). The choice to adopt technology in teaching is one example of teachers’ innovativeness, which has scarcely been examined along with other pedagogical characteristics.

In addition to innovativeness, other potentially fruitful variables might not have been considered yet, such as teachers’ learning patterns. Learning patterns refer to teachers’ differences in the way they learn, and the patterns are comprised of beliefs, motivation and activities usually employed while learning. Research has shown that teachers vary in their approaches to learning when they respond to educational changes (Vermunt & Endedijk, 2011). Recent evidence (Murtonen et al., submitted) suggests that teachers’ adaptive learning patterns can be fostered by pedagogical training which is viewed as a tool to help teachers think beyond the given context (McAleavy et al., 2018).

It is worth noting that studies thus far have addressed the role of *post*-COVID-19 pedagogical training (e.g. Schildkamp et al., 2020) offered to teachers, while little is known about the role of *pre*-COVID-19 training. Examining the role of pre-COVID-19 training is crucial because if it is found to be effective, HEIs could avoid the need for rushed trainings. On the other hand, if it is ineffective, this could indicate a weakness in the current training methods that were unable to assist teachers during unexpected situations. Furthermore, teachers in HEIs are not a homogeneous group in relation to their readiness and ability to cope with online teaching (Scherer et al., 2021). Differences are assumed to exist in terms of teaching experience and discipline. Thus, for the current study, we set two research objectives: (1) to profile teachers based on their digital innovativeness and the extent to which they implemented changes to adapt their teaching practices to the COVID-19 restrictions, and (2) to explore how teachers’ learning patterns, previous pedagogical training, teaching experience and discipline are associated with the resulting teacher profiles.

## Theoretical framework

### Adaptation to COVID-19 and digital innovativeness in teaching

The COVID-19 pandemic has suddenly accelerated the process of digitalizing teaching and learning, leading to a mixed experience with both positive and negative outcomes (Nicklin et al., 2022). According to Hadar et al. (2020), the pandemic has increased the workload for teachers who may not have been prepared with the necessary social-emotional competencies. Meanwhile, Spoel et al. (2020) found that a lack of interaction, time pressure, and overload were the most expected and experienced negative aspects of online teaching. This was echoed by Mittal et al. (2021) who added that the challenges faced by teachers during the early days of the pandemic included a lack of experience, difficulty teaching technical courses, and concerns about students' readiness for online learning. In contrast, Marek et al. (2021) found that university teachers' experiences varied between positive and negative, with teachers expressing higher levels of workload and stress in online teaching compared to face-to-face teaching, but also highlighting the positive aspects of learning, such as the need for adaptability and good planning. Moreover, Meishar & Ariella (2021) investigated the emotions experienced by university lecturers and found that success was the most strongly experienced emotion, followed by opportunity.

A combination of factors related to the context (institutional) and the teacher (psychological) contribute to these positive and negative experiences. For instance, the support offered by HEIs to teachers in terms of providing the needed infrastructure, facilitating access to technologies and organising specialised pedagogical training opportunities are among the institutional promoters of positive online teaching and learning experiences; however, these institutional means cannot succeed unless the teachers themselves possess innovativeness and show the willingness to continuously learn, embrace change, experiment with new ways of intertwining technology in teaching and seek the best ways to harness digital tools for educational purposes.

Innovativeness has been repeatedly cited as a crucial characteristic to predict teachers’ reactions towards change and newness (Aldahdouh et al., 2020). Innovativeness is defined based on three approaches: behavioral, domain-specific and general (Goldsmith & Foxall, 2003). The general approach defines innovativeness as a deep construct or a psychological characteristic that shapes the individual disposition towards innovations in general; however, for the behavioral approach, innovativeness is considered the actual act of adopting innovations (i.e. actualised innovativeness; Midgley & Dowling, 1978). Because individuals show varied tendencies and interests in relation to different kinds of innovations, the domain-specific innovativeness approach has been introduced to understand the individual tendency to adopt innovation within a specific domain, such as in teaching. It is worth noting that actualised innovativeness is always domain-specific. In this study, we were mainly interested in university teachers’ actualised innovativeness in the domain of adopting technology for teaching (which will be referred to hereafter as digital innovativeness).

Two main methods have been described in the literature to measure actualised innovativeness: the time-of-adoption and cross-sectional methods (Goldsmith & Foxall, 2003). The time-of-adoption method relies on the notion of the *earliness* of innovation adoption. In other words, the respondents are asked to recall and indicate when they started the adoption of a specific innovation. The earlier one adopts the innovation, the more innovative he/she is. The cross-sectional method relies on the notion of the *versatility* of adopting innovations within a specific domain. In this case, respondents are asked to select which innovations they have adopted from among a comprehensive list of innovations within a certain category. The more innovations one has embraced, the more innovative the individual. For the current study, we adopted the cross-sectional method to measure the teachers’ digital innovativeness to overcome the recall problem associated with the time-of-adoption method; however, the cross-sectional method has been criticised for not being able to distinguish between a participant who started using the technology only recently (a late adaptor) and a participant who was among the first to acquire it (an innovator), and thus a certain amount of variation between participants is lost (Goldsmith & Foxall, 2003).

Investigating teachers’ adoption of technology in higher education is currently a vibrant research topic (Aldahdouh et al., 2020; Liu et al., 2020; Mercader & Gairín 2020; Mittal et al., 2021; Sailer et al., 2021) for several reasons. For example, recent studies have shown that teachers lag behind their students in technology usage, especially at the university level (Mercader & Gairín, 2020), their adoption of technology often does not meet the HEIs’ aspirations (Aldahdouh et al., 2020; Liu et al., 2020) and there is still ambivalence surrounding their integration of digital technology in teaching (Sjöberg & Lilja, 2019). This gap has led to a growing, yet inconclusive, line of research that aims to propose models of factors contributing to higher education teachers’ adoption of technology (Liu et al., 2020; Martin et al., 2020; Sailer et al., 2021). For instance,Sailer et al. (2021) proposed the Cb-model in which they assumed that university teachers’ digital technology usage is influenced by institutional and individual factors. Institutional factors include infrastructure, technical and educational support and digitalisation policy, whereas individual factors include teachers’ knowledge, basic and technology related teaching skills, attitudes and qualification regarding teaching and learning with digital technologies.

The factors related to teachers’ technology adoption have been explored in light of more broad technology acceptance theories, such as diffusion of innovation (Rogers, 2003) and the technology acceptance model (Liu et al., 2020). According to these theories, innovators usually have personal characteristics that enable them to be change agents. For instance, research has shown that innovativeness is associated with higher levels of personal initiative (Miron et al., 2004), self-efficacy (Vinarski-Peretz et al., 2011), openness to experience (Ali, 2018) and mastery goal orientation and a lower level of performance avoidance goal orientation (Aldahdouh et al., 2019). These qualities enable innovators to take the initiative to disrupt the status quo and to find novel ways to perform tasks when facing a challenging situation. For example, when investigating the factors that contribute to blending learning adoption in higher education, Bruggeman et al. (2021) concluded that realizing a need for change and daring to experiment (and fail) were found as crucial teacher attributes.

### Teachers’ learning patterns, prior pedagogical training, teaching experience and discipline

In their recent study, Vermunt et al. (2019) established three qualitatively different teacher learning patterns, which refer to coherent wholes of learning activities, beliefs about learning and motivation for learning in a certain period of time (see also Bakkenes et al., 2010). The first—the meaning-oriented learning pattern—describes an approach where the teacher tries to understand, for instance, how students learn, why they do not understand something and why certain teaching methods are effective. Furthermore, the teacher tries to see how different lessons relate to each other. Thus, meaning-oriented teachers try to understand the logic behind their teaching actions, student learning and the approach to learning rather analytically.

The second pattern, application-oriented learning, represents a more practical orientation to learning (Vermunt et al., 2019). Typical for this type of learning is that teachers seek new tips and ideas for their teaching to apply them in practice. Rather than trying to understand why certain teaching methods are effective, they want to know which teaching methods are effective. These teachers feel that they learn most from their own practical experiences. Their actions are motivated by the immediate improvement of their teaching practices.

The third pattern, problematic learning, relates to teachers who struggle with educational development, experience discrepancies between how they teach and how they want to teach and do not know how to teach in another way than they do (Vermunt et al., 2019). For this learning pattern, negative feelings towards teaching are typical.

Previous research has shown that the more favourable learning patterns of meaning and application orientation are associated with adaptive consequences as opposed to problematic learning, which is associated with maladaptive consequences (Murtonen et al., submitted; Vermunt et al., 2019; Vermunt & Endedijk, 2011). Thus, we assume that positive learning patterns would be associated with coping with the emergency remote teaching requirements caused by COVID-19.

In the current digital age, all educators are expected to possess a minimum level of digital skills; however, the issue not only involves mastering digital skills but also acquiring pedagogical skills. To teach effectively in digital environments, a teacher requires a pedagogical understanding of how to apply digital skills. The topics of typical teaching enhancement training include, for example, pedagogy and didactics, student-centered learning and assessments of learning outcomes (Gaebel et al., 2018). Concerning digitally enhanced learning, topics related to use of technology seem to be more frequent than the related pedagogies (i.e., how to teach with information and communications technology; ICT). Although pedagogical training tends to include some basic knowledge and skills concerning the pedagogy of digital learning, this might not be enough. To apply new technologies effectively, teaching staff may need ongoing, relevant and up-to-date training (Chircop et al., 2020).

Previous research on the impact of pedagogical training in higher education is contradictory, but overall, it seems that pedagogical training has positive yet sometimes rather small effects (see e.g. Stes et al., 2012; Teräs, 2016; Trigwell et al., 2012; Vilppu et al., 2019). Successful professional development programmes seem to be collaborative, reflective and of long duration (Teräs, 2016); however, shorter programmes have also resulted in positive developments, at least among novice teachers (Vilppu et al., 2019; Ödalen et al., 2018). In short, research shows that teachers’ conceptions of teaching and approaches to teaching can be developed through pedagogical training (e.g. Vilppu et al., 2019; Postareff et al., 2007).

Regarding the relationship between experience and expertise in teaching, it seems be a sort of double-edged sword. On one hand, one might think that practice makes perfect, and thus experience is solely a positive aspect. On the other hand, experience and any kind of practice do not straightforwardly lead to a higher level of expertise; rather, ‘deliberate” practice is needed (Ericsson & Pool, 2016). Furthermore, teaching experience might sometimes even act as an obstacle for development. For instance, several studies show that changing conceptions of teaching among experienced teachers may be challenging (e.g. Ertmer, 2005; Lueddeke, 2003; Marsh, 2007; Postareff & Nevgi, 2015). In addition to the ambiguous role of teaching experience on teachers’ learning and development, its impact on technology adoption remains unclear (Aldahdouh et al., 2020). While some studies have shown that less-experienced teachers tend to accept technology better than more-experienced teachers (Zalat et al., 2021), other studies have proposed the opposite (Sailer et al., 2021).

For discipline, research to date has yielded inconclusive findings regarding the effect of discipline on technology adoption (Aldahdouh et al., 2020; Mercader & Gairín, 2020). Although research on university staff generally indicates that those who work in soft disciplines (e.g., history) tend to embrace new innovations more than their counterparts who work in hard disciplines (e.g., physics; Manca & Ranieri, 2016; Nández & Borrego, 2013; Wang & Meiselwitz, 2015), studies on university teachers in particular (Mercader & Gairín, 2020) have shown that those in the arts and humanities seem to perceive greater obstacles to using digital technology than other disciplines. Thus, a continued analysis of the roles previous pedagogical training, teaching experience and discipline play in coping with changes in teaching during COVID-19 remains justified.

## Methods

### Research instrument and sample

The data were collected through an online self-reported questionnaire​ developed on Microsoft Office 365 Forms. The questionnaire link was distributed via email to all teachers and supervisors of an HEI in Finland in April of 2020. All respondents provided informed consent on the first page on the questionnaire.

The questionnaire consisted of three sections. The first included questions about participants’ background information and participation in previous pedagogical training. The second section consisted of two open-ended questions about online teaching and digitalisation before and during COVID-19. The last section included items from the Inventory of Teacher Learning devised by Vermunt et al. (2019). The inventory is comprised of three subscales: meaning-orientated learning (14 items; e.g. ‘I try to understand why certain teaching methods work’), application-oriented learning (nine items; e.g. ‘I want to apply new ideas in my teaching’) and problematic-oriented learning (nine items; e.g. ‘I only want to learn things that I can use immediately in my teaching’). A Likert scale was used that ranged from 1 (completely disagree) to 5 (completely agree). The items were translated into the Finnish language and underwent a formal back-translation before being published. The participants were offered the choice to answer either in English or in Finnish.

A total of 381 responses were received, of which 378 were deemed valid and used for the analysis. The participants reported their teaching experience as follows: 45 (12%) had less than two years of experience, 59 (16%) had from two to less than five years of experience, 58 (15%) had from five to less than 10 years of experience, 73 (19%) had from 10 to less than 15 years of experience and 143 (38%) had 15 or more years of experience. The majority (n = 303, 80%) of the participants had attended some pedagogical training or courses, and 31% of participants reported having over 301 h of teaching/supervision annually. The sample included participants from almost all university faculties, and thus the sample was categorised according to Biglan’s (1973) classification into two academic disciplines: hard disciplines such as physics and chemistry (*n* = 166, 44%) and soft disciplines such as history and economics (*n* = 212, 56%).

### Analysis

The teacher learning patterns instrument was tested to validate its factorial structure using Structural Equation Modelling, as described in a previous study (Murtonen et al., submitted). The results indicated that the scales were valid and reliable. We checked the normality of the data against cut-off values of less than two for skewness and less than seven for kurtosis (Kim, 2013). No violations in the normality assumption were detected. Table 1 shows the means, standard deviations, Cronbach Alpha values and distributional properties (skewness and kurtosis).

**Table 1:** Means, standard deviations, Cronbach Alpha values, skewness and kurtosis of the study variables

Study variable	M (SD)	Skewness (S.E)	Kurtosis (S.E)	Alpha Cronbach
Meaning-oriented learning pattern	3.95 (0.535)	-0.526 (0.125)	0.736 (0.250)	0.82
Application-oriented learning pattern	4.38 (0.474)	-0.918 (0.125)	1.657 (0.250)	0.74
Problematic-learning pattern	2.04 (0.630)	0.407 (0.125)	-0.033 (0.250)	0.61

To analyse the two open-ended questions, a quantitative content analysis was performed by two researchers (see Table 2). The coding scheme for the first question was derived from the Diffusion of Innovation theory (Rogers, 2003). In particular, we employed the cross-sectional method to classify the participants into two main categories: avoiders and embracers. The avoiders are those who did not show versatility in using technological tools in teaching before COVID-19, while embracers are at opposite extreme. The coding scheme for the second question was derived inductively from the teachers’ answers (Elo & Kyngäs, 2008), which resulted in two categories: survival, and ambitious adapters. Survival adapters are those who report at max one change in response to COVID-19, while ambitious adapters are at opposite extreme.

**Table 2:** Content analysis coding scheme

Question	Code	Criteria	Example
*1. Describe how you have utilised digital opportunities in your teaching/supervision before the COVID-19 situation? For example, in which subjects and what kind of tools and learning environments have you used?*	1 = ‘Avoiders’	None or only one technological application or tool used with no details explaining how it was used Or, when the participants vaguely mention that they used some digital tools but did not explain what the tools are or how they employed them in teaching	‘*Very little in the way of teaching: mainly Moodle and email. Same for supervision*’. Teacher 59
	2 = ‘Embracers’	Multiple technologies used with a richer explanation	‘*I have been teaching entirely online since 2000. In addition to e-learning environments (WebCT, later Moodle), I have used all sorts of other things, such as screen capture videos, social media environments, lecture capture software (ECHO 360), various content production applications, web conferencing software, *etc*. In the early years when applications were scarce, I used to create content myself using html and JavaScript… My pedagogy is socio-constructivist. The content has been all educational and language content*’. Teacher 29
*2. Due to COVID-19, all teaching/supervision needed/needs to be suddenly transformed to distance mode. What kind of changes has this caused in your teaching/supervision? For example, what kind of pedagogic solutions and/or tools have you implemented in different subjects or courses?*	1 = ‘Survival adapters’	None or only one instructional change adapted but with no details explaining what or how the changes were implemented	‘*Virtual lectures *via* Zoom, small group work and mentoring meetings *via* Teams*’. Teacher 65
	2 = ‘Ambitious adapters’	Multiple instructional changes adapted with a richer explanation	‘*I have used Moodle more systematically to give instructions, provide areas for discussion and feedback, *etc*. I have obviously been teaching using Zoom and have used all the key features, such as breakout rooms, screen sharing and recording. I have also used screen recording software to give feedback on academic writing and presentation performances. In addition, I have used software such as Padlet*’*.* Teacher 96

The two researchers coded the data separately, and the inter-rater reliability was examined by calculating the Kappa coefficient and percental agreement (Syed & Nelson, 2015). When there was disagreement about any content, uncertainty was resolved by examining the coding again independently. Initially, the Kappa values were 0.760 (83.03% agreement) and 0.742 (81.95% agreement) to the first and second questions, respectively. These results indicate an acceptable rate of consistency between the two coders (over 0.700 according to Syed & Nelson, 2015). Finally, coding was examined once more to increase the validity. This last coding was done by one researcher after eight months elapsed from the first coding, and the Kappa values were 0.949 (96.68% agreement) to the first question and 0.950 (98.44% agreement) to the second question.

**Fig. 1:**
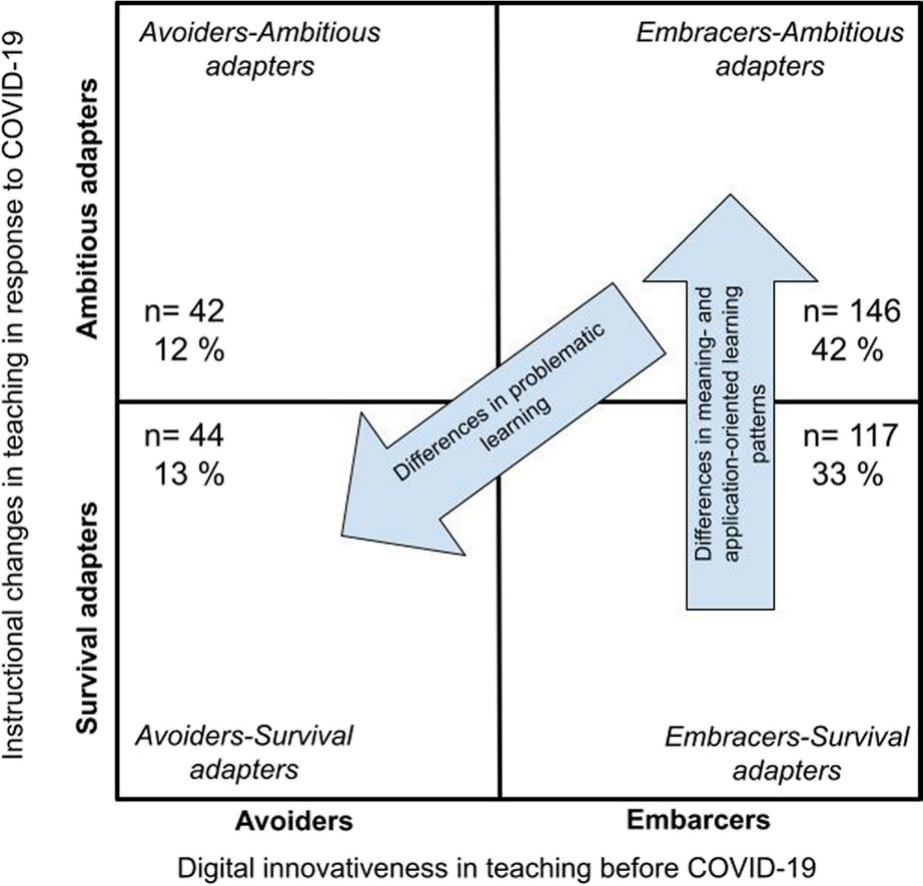
Teacher groups. Note: n = 349. All cases that did not provide answers to the questions were excluded

The coding scheme represents two dimensions: one is the degree of digital innovativeness, and the other is the degree of adaptation to COVID-19. Crossing these two dimensions resulted in four quadrants: Avoider Survival Adapters, Avoider Ambitious Adapters, Embracer Survival Adapters and Embracer Ambitious Adapters (see Fig. 1).

We performed the Chi-squared test using SPSS (version 26) software to examine whether these groups are significantly associated. The findings revealed a significant association among these four groups (χ^2^(4) = 139.93, *p* < 0.001, Cramer’s V = 0.43). The participants who had not been categorised due to missing answers were excluded from further analyses. The Kruskal–Wallis test was performed to examine whether there were significant differences among the groups in learning patterns. We opted to use a non-parametric test over the analysis of variance because the sizes of the teacher groups are unequal, and when the assumption of the homogeneity of variance is violated, a non-parametric test for multiple independent samples is recommended (Rosenthal, 2012). Chi-squared tests were utilised to study the association between teacher groups and previous pedagogical training, teaching experience and discipline.

## Results

### Teachers’ profiles in the four groups and the association with teacher characteristics

Table 3 shows the teacher profiles in the four groups based on their previous pedagogical training and teaching experience. A chi-squared test was performed to examine the extent to which participating in previous pedagogical training is associated with belonging to the teacher groups. The findings showed a significant strong association (χ^2^(3) = 14.71, p < 0.01, Cramer’s V = 0.205), suggesting that teachers who did not have previous pedagogical training tended to belong to the Embracer Survival Adapters group, while teachers who attended pedagogical training previously were more likely to belong to the Embracer Ambitious Adapters group.

**Table 3:** Teachers’ profiles in the four groups

		Avoider Survival Adapters	Avoider Ambitious Adapters	Embracer Survival Adapters	Embracer Ambitious Adapters	All samples
N (%)		44(13%)	42(12%)	117(33%)	146(42%)	349(100%)
Discipline	Hard	28	19	**56**	49	152
	Soft	16	23	61	**97**	197
Pedagogical training	No	15	7	**25**	15	62
	Yes	29	35	92	**131**	287
Teaching experience	Less than 2 years	**12**	6	**12**	8	38
	2 – less than 5 years	7	8	19	**22**	56
	5 – less than 10 years	7	7	15	**24**	53
	10 – less than 15 years	8	8	19	**36**	71
	15 years or more	10	13	52	**56**	131

Bolded values indicate statistically significant differences (*p* < 0.05) between groups

We then tested the extent to which teaching experience is associated with belonging to teacher groups. The results of the Chi-squared test indicated a significant moderate association (χ^2^(3) = 8.22, p < 0.05, Cramer’s V = 0.154). Teachers with less than two years of experience tended to belong to the Avoider Survival Adapters group and Embracer Survival Adapters group. Teachers with longer teaching experience (two years or more) tended to belong to the Embracer Ambitious Adapters group.

In addition, the Chi-squared results indicated a strong significant association between teacher groups and discipline (χ^2^(3) = 14.08, *p* < 0.01, Cramer’s V = 0.201). In other words, the finding suggested that teachers working in hard disciplines were more likely to belong to the Embracer Survival Adapters group, while teachers working in soft disciplines were more likely to belong to the Embracer Ambitious Adapters group.

### Association between teacher groups and learning patterns

The results of the Kruskal–Wallis test indicated that the teacher groups were significantly different from one another in their learning patterns, as shown in Table 4.

**Table 4:** Kruskal–Wallis test to compare teacher groups and learning patterns

	Meaning learning pattern	Application learning pattern	Problematic learning pattern
Test statistic	10.878 (*df* = 3)	10.367 (*df* = 3)	11.483 (*df* = 3)
Asymptotic sig	0.012*	0.016*	0.009*
*Mean rank*			
Avoider Survival Adapters (1)	159.10	149.88	215.33
Avoider Ambitious Adapters (2)	164.18	175.83	177.40
Embracer Survival Adapters (3)	158.82	160.60	179.80
Embracers Ambitious Adapter (4)	195.87	193.87	158.31
*Pairwise comparison*			
(1)-(2)	*p* = 1.000	*p* = 1.000	*p* = 0.475
(1)-(3)	*p* = 1.000	*p* = 1.000	*p* = 0.269
(1)-(4)	*p* = 0.202	*p* = 0.064	*p* = 0.006*
(2)-(3)	*p* = 1.000	*p* = 1.000	*p* = 1.000
(2)-(4)	*p* = 0.433	*p* = 1.000	*p* = 1.000
(3)-(4)	*p* = 0.018*	*p* = 0.045*	*p* = 0.502

* *p* < 0.05; *p*-values were adjusted using the Bonferroni correction for multiple tests

The pairwise comparison tests revealed that Embracer Ambitious Adapters have significantly lower problematic learning patterns than Avoider Survival Adapters. Moreover, Embracer Ambitious Adapters were found to have significantly higher meaning-oriented and application-oriented learning patterns than Embracer Survival Adapters.

## Discussion

The aim of this study was to advance our understanding of the factors that contributed to university teachers’ ability to implement instructional changes to cope with COVID-19.

By examining digital innovativeness and the extent to which teachers applied changes in their pedagogical practices, we were able to categorise teachers into four groups (Fig. 1) and to closely analyse the differences among the groups in terms of pedagogical tendencies (i.e. learning patterns) and career background (previous pedagogical training, teaching experience, discipline). The juxtaposition of the contrasting characteristics (i.e., Avoider vs. Embracers and Survival Adapters vs. Ambitious Adapters) revealed teachers’ trajectories in their response to COVID-19. In what follows, we present the characteristics of each group along with the differences between them according to the study variables.

The first group, labelled Avoider Survival Adapters, refers to less innovative teachers who avoid newness, resist change and react cautiously when they encounter something novel. Consequently, they minimally adapted their teaching practices when they were forced to shift to online teaching due to the pandemic. The Avoider Ambitious Adapters group, on the other hand, includes less innovative teachers who avoid newness and resist change, yet they surprisingly reacted differently when they were faced with COVID-19 restrictions. The Embracer Ambitious Adapters group refers to innovative teachers who were capable of reacting fast and implementing multiple changes to adapt their teaching properly in the early stages of COVID-19. Figure (1) shows that the largest number of teachers in the sample belongs to this group, which could represent the optimal target because it includes the case in which teachers possess an adaptive psychological characteristic (i.e., innovativeness) and adaptive expertise (i.e., respond adequately to a crisis, such as COVID-19). The Embracer Survival Adapters group refers to innovative yet slowly respondent teachers. Despite their innovativeness, it seems they lacked the initiative to delve into unfamiliar teaching settings and practices.

To further understand the characteristics of these groups, we examined the differences among them in terms of learning patterns. The findings indicated that Embracer Ambitious Adapters scored significantly lower than Avoider Survival Adapters for the problematic learning pattern. This result reflects those of Vermunt et al. (2019), who also reported that teachers with problematic learning patterns often avoid innovations and experience negative emotions as a result of friction between what they want or expect and how teaching occurs in practice. These teachers feel safe when they avert learning about new teaching methods. Vermunt and Endedijk (2011, p. 297) indicated that, ‘teachers adopting a struggling approach hardly did experiments (any more), but thought and worried a lot while fighting or giving in to the tendency to fall back into their old routine practices’.

It is interesting to note from the findings that although both possess innovativeness, Embracer Ambitious Adapters seemed to be more meaning- and application-oriented than Embracer Survival Adapters. This result implies that meaning-oriented and application-oriented learning approaches are essential for teachers to apply instructional changes. An explanation for this could be that application-oriented teachers by definition are characterised by the tendency to apply new methods and to experiment with new ideas. According to Vermunt et al. (2019), these teachers heavily apply practices, yet they stick within the boundaries of their existing theory of practice. In contrast, meaning-oriented teachers tend to expand their understanding of new practices and to implement changes based on their understanding, thus extending the boundaries of their existing theory of practice (Vermunt et al., 2019). As meaning-directed teachers by nature seek to reflect on how and why a specific change in pedagogical practices would lead to better learning outcomes, Bakkenes et al. (2010) found that this learning approach would be much more adequate for teachers when it comes to educational innovations. Previous studies (Oosterheert et al., 2002) that have examined teachers’ personality variables associated with meaning-oriented learning revealed some related yet different variables than innovativeness, such as self-esteem, extraversion, emotional stability and tolerance of ambiguity.

The most obvious finding to emerge from the current study is the role of pre-COVID-19 pedagogical training. The findings showed that teachers who did not have previous pedagogical training tended to belong to the Embracer Survival Adapters group, while teachers who had attended pedagogical training previously were inclined to belong to the Embracer Ambitious Adapters group. These results are in agreement with a previous study (Oliveira et al., 2021), which showed that pedagogical training helps teachers confidently tailor their teaching practices to meet new demands. A synthesis of the research on the characteristics of effective professional development has highlighted the importance of enabling ‘contextualisation by teachers to their particular teaching situations’ (McAleavy et al., 2018, p. 12), which in turn assists teachers in adapting their pedagogical practices when they teach in varied contexts.

The present study has provided further evidence of the association between teacher groups and their background information and has shown that teachers in both hard and soft disciplines differ in terms of the extent to which they apply changes, albeit both seem to be innovators. Unlike the study by Lee & Jung (2021), which showed no differences in teachers’ instructional changes in terms of course discipline, the current research indicated that academics teaching soft subjects seem to apply changes more often than their counterparts who teach hard subjects. A possible explanation for this finding might be connected to teaching approaches and the extent to which teachers emphasise deep approaches to student learning. Previous research has shown that teachers in natural sciences are more content-focused in their teaching in contrast to their colleagues in the humanities, who are more learning-focused (Lueddeke, 2003) and place greater emphasis on deep learning. In other words, teachers’ pursuit of supporting student-centered teaching could be the reason behind their extensive implementation of instructional changes during COVID-19. For example, a tutor teaching history may implement flipped classroom with multiple online learning activities whereas a tutor teaching physics may solely use online whiteboard in explaining equations together with the course slides used in teaching before COVID-19.

In terms of teaching experience, the results showed that teachers with less than two years of experience tended to adapt their teaching less extensively than their counterpart teachers with more teaching experience (two years or more). One possible explanation is that less-experienced teachers may not yet be equipped with the digital pedagogical competences needed to confidently apply changes to their teaching practices. Sailer et al., (2021, p. 7) stated that ‘for teachers in higher education, their experience during their own studies as well as during the early phases of their academic careers can have an impact on the way they will later use digital technology themselves in their teaching’.

## Implications

While COVID-19 has constituted a radical and unprecedented challenge for most university teachers worldwide, we nevertheless sought to consider these conditions as lending themselves to informing HEIs’ practitioners, scientists and policy makers. First, HEIs should take concrete actions to promote university teachers’ innovativeness and willingness to change. Changes are occurring faster than ever. Keeping pace with these changes and responding effectively means that teachers should tolerate ambiguity and should have a sense of curiosity towards new ideas; however, innovativeness is not enough. The way teachers go in their learning has been proven to be essential as well.

This study has raised important questions regarding the nature of teachers’ learning patterns and how they can be beneficial in coping with challenges. For an innovative teacher, a meaning and application orientation to learning appeared to be necessary to adjust teaching and to implement instructional changes. As teachers with meaning orientation learning patterns tend to apply changes based on understanding and a thoughtful plan, fostering meaning-directed learning should be a priority of HEIs seeking to adapt to the ever-changing world. On the other hand, problematic learning should be overcome due to its negative consequences. HEIs should help teachers who experience ambiguity regarding how to teach in a new way or who feel confused about how to experiment with new ideas. A rapid intervention at the right time helps teachers overcome obstacles and pushes them forward.

The current study adds to the growing body of literature that indicates the significance of pedagogical training (Aldahdouh et al., 2022; Ödalen et al., 2018; Vilppu et al., 2019). What distinguishes this study from others is that we have examined the value of previous pedagogical training and have provided evidence that pedagogical training helped innovative teachers embrace more changes in their teaching practices during the COVID-19 pandemic. Although the training does not necessarily include specific guidelines regarding how to deal with emergencies, it can be considered a way to develop teachers’ adaptive expertise and to unlock their potential to think unconventionally.

## Limitations and future research

Despite the strong results, questions regarding the Avoider Ambitious Adapters group remain unanswered. This group of teachers seemed to lack innovativeness, yet they coped with COVID-19 by implementing several changes to adapt their teaching to the pandemic context. It may be that the pre-COVID digital solutions were not appealing to them. Due to the small number of participants in this group, the current study felt short in providing evidence of the association between Avoider Ambitious Adapters group, learning patterns and teacher demographics. Further research should be conducted to investigate the drivers for this group to respond to COVID-19 in such a way and to examine factors other than those analysed in the current study. Another limitation is that the changes made by teachers were examined quantitively to facilitate categorising teachers into groups. Future studies should consider a within-subject design, which allows for capturing the type of change adaptations that are implemented and which strategies are used in resolving challenges.

Taken together, the COVID-19 pandemic offered opportunities for teachers to revivify teaching in new ways, and their experiences can be exploited in the post COVID-19 era we are currently experiencing.

## Data Availability

The data that support the findings of this study are available on request.
